# Range‐wide breeding habitat use of the critically endangered Yellow‐breasted Bunting *Emberiza aureola* after population collapse

**DOI:** 10.1002/ece3.7668

**Published:** 2021-05-18

**Authors:** Ilka Beermann, Alexander Thomas, Yury Anisimov, Marc Bastardot, Nyambayar Batbayar, Batmunkh Davaasuren, Yury Gerasimov, Makoto Hasebe, Gleb Nakul, Jugdernamjil Nergui, Pavel Ktitorov, Olga Kulikova, Wieland Heim

**Affiliations:** ^1^ Institute of Landscape Ecology University of Münster Münster Germany; ^2^ EuroNatur Foundation Radolfzell Germany; ^3^ School of Environmental Science and Engineering Southern University of Science and Technology Shenzhen China; ^4^ Baikalsky State Nature Reserve Tankhoy Russia; ^5^ Department of Ecology and Evolution University of Lausanne Lausanne Switzerland; ^6^ Wildlife Science and Conservation Center Ulaanbaatar Mongolia; ^7^ Kamchatka Department of Pacific Geographical Institute Far‐eastern Branch of Russian Academy of Science Petropavlovsk‐Kamchatsky Russia; ^8^ Sarobetsu Eco‐Network Toyotomi Japan; ^9^ Institute of Biology of Komi Science Centre of the Ural Branch of the Russian Academy of Sciences Syktyvkar Russia; ^10^ Institute of Biological Problems of the North FEB RAS Magadan Russia; ^11^ Birds Russia, Sakhalin Branch Yuzhno‐Sakhalinsk Russia; ^12^ University of Konstanz Konstanz Germany

**Keywords:** distribution, Japan, Mongolia, predict, Russia, vegetation

## Abstract

The population of the Yellow‐breasted Bunting *Emberiza aureola*, a formerly widely distributed and abundant songbird of northern Eurasia, suffered a catastrophic decline and a strong range contraction between 1980 and 2013. There is evidence that the decline was driven by illegal trapping during migration, but potential contributions of other factors to the decline, such as land‐use change, have not yet been evaluated. Before the effects of land‐use change can be evaluated, a basic understanding of the ecological requirements of the species is needed. We therefore compared habitat use in ten remaining breeding regions across the range, from European Russia to Japan and the Russian Far East. We also assessed large‐scale variation in habitat parameters across the breeding range. We found large variation in habitat use, within and between populations. Differences were related to the cover and height of trees and shrubs at Yellow‐breasted Bunting territories. In many regions, Yellow‐breasted Buntings occupied early successional stages, including anthropogenic habitats characterized by mowing, grazing, or fire regimes. We found that the probability of presence can be best predicted with the cover of shrubs, herbs, and grasses. Highest probabilities were found at shrub cover values of 40%–70%. Differences in habitat use along a longitudinal gradient were small, but we found strong differences across latitudes, possibly related to habitat availability. We conclude that the remaining Yellow‐breasted Bunting populations are not limited to specific habitat types. Our results provide important baseline information to model the range‐wide distribution of this critically endangered species and to guide targeted conservation measures.

## Graphical abstract

We compared habitat use of the critically endangered Yellow‐breasted Bunting in ten remaining breeding regions across the range and assessed large‐scale variation in habitat parameters. We conclude that the remaining Yellow‐breasted Bunting populations are not limited to specific habitat types. Our results provide important baseline information to model the range‐wide distribution of this threatened species.

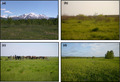

## 1 INTRODUCTION

Key to the conservation of species is an understanding of their natural history, including the habitats they require for breeding and foraging (Walters, 1991). Species‐specific habitat suitability is expected to gradually change from prime habitats in the center of distribution toward secondary habitats at the limits of the distribution (Brown, 1984; Brown et al., 1995). However, in many critically endangered species, only peripheral populations have persisted (Channell & Lomolino, 2000). These peripheral sites might have represented suboptimal habitats in the past but have sustained populations during human‐caused extinctions due to their isolated position (e.g., islands, mountain ranges) (Channell & Lomolino, 2000).

A significant range contraction was observed recently in the Yellow‐breasted Bunting *Emberiza aureola,* a formerly superabundant and widely distributed Palearctic songbird (Kamp et al., 2015). Its breeding range stretched from Finland in the west to Kamchatka in the east and from northern Mongolia up to the polar circle (BirdLife International, 2020). The species likely originates from East Asia, the diversification hotspot of *Emberiza* buntings (Päckert et al., 2015), and colonized peripheral areas west of the Ural Mountains during the 19th century (Mischenko, 2019). Between 1980 and 2013, the population declined by 84.3%–94.7%, and the western range limit retracted 5,000 km to the east (Kamp et al., 2015). This precipitous decline was linked to unsustainable harvest rates during the nonbreeding season in China and Southeast Asia (Chan, 2004; Heim et al., 2021; Kamp et al., 2015), but the role of additional limiting factors such as habitat loss is unknown. The western subspecies *E. a. aureola* has been suggested to be more specialized with regard to breeding habitat use compared with the eastern *E. a*. *ornata* (Glutz von Blotzheim & Bauer, 1993).

Yellow‐breasted Buntings occupy a wide range of habitats during the breeding season, including bogs, meadows, mountain tundra, forest steppe, broadleaf forests, open conifer forests and clearings, dwarf bamboo shrubs, and, on arable land, hay meadows and abandoned fields close to villages, from lowlands to an altitude of more than 2,000 m (Dement'ev & Gladkov, 1954; Glutz von Blotzheim & Bauer, 1993; Nakamura et al., 1968; Radde, 1862; Rogacheva, 1992). Highest abundances are reached in wet lowland meadows dominated by tall forbs or shrubs in river valleys (Glutz von Blotzheim & Bauer, 1993). Little is known about the habitats currently used by persisting or recolonizing populations. Breeding Yellow‐breasted Buntings have recently been reported from river valleys near Irkutsk (Ivushkin, 2017) and from wet grasslands with willow shrubs in the Tunka Valley (Drillat, 2019) and the Zeya‐Bureya Plain (Richter et al., 2020) in Russia. These sites have likely sustained populations before and after the decline. However, no data from peripheral populations and on larger spatial scales are available, which could be used to inform conservation schemes for this critically endangered species (Whittingham et al., 2005).

We aimed to (a) describe the habitat use of the critically endangered Yellow‐breasted Bunting across its range, (b) to assess which environmental parameters are most important for habitat use of the species, and (c) to examine variation in habitat use along latitudinal and longitudinal gradients.

## 2 METHODS

### 2.1 Fieldwork

We compiled data from a network of collaborators that had monitored Yellow‐breasted Buntings during the breeding season (May to July) in 2017, 2018, and 2019, following the report of strong declines in 2015 (Table 1). All contributors searched for singing males and used the individual song post as the center of a 10‐by‐10 meters plot for habitat mapping (“presence plots”). We visually estimated the total vegetation cover [in %] and the cover [in %] of trees, shrubs, dwarf shrubs, grass, herbs, and litter by standing at each of the four corners of the plot. We defined trees as a woody plant with a single trunk and a shrub as a woody plant with more than one trunk. Low‐growing shrubs (<1 m height) were defined as dwarf shrubs (e.g., *Salix myrtilloides*). Reed was classified as grass. Woody parts of *Artemisia* were classified as herbs. If burned and dead parts were still attached to a plant, they were treated as part of it. We estimated the mean height [in cm] of trees, shrubs, dwarf shrubs, grass, herbs, and litter. Additionally, the cover of bare soil [in %] was estimated. Furthermore, we noted signs of fire, grazing, or mowing from the current breeding season (0 = no, 1 = yes). Moisture was estimated in four categories (0 = completely dry, 1 = moist or wet, 2 = waterlogged, and 3 = standing open water or flooded soil).

**TABLE 1:** Study regions from east to west, with sample sizes (*N*) for investigated Yellow‐breasted Bunting presence plots (P) and corresponding pseudo‐absences (A)

Study area	Country	Coordinates	*N*_P_	*N*_A_	Habitats
Chukotka (Markovo Village)	Russia	64°41′N 170°25′E	12	0	Grasslands with willow trees
Kamchatka (various sites)	Russia	54°38′N 158°33′E	190	31	Wetlands, forests
Hokkaido (Sarobetsu Plain)	Japan	45°7′N 141°41′E	11	0	Wetlands
North Sakhalin (Volchinka River)	Russia	53°24′N 142°30′E	12	0	Grasslands
Central Sakhalin (Poronaysk Town)	Russia	49°14′N 143°5′E	2	0	Grasslands with willow trees
Amur (Muraviovka Park)	Russia	49°55′N 127°40′E	162	250	Wetlands, fallow land
Khurkh Valley	Mongolia	48°32′N 110°32′E	14	10	Wetlands
Baikal (Kabansky Zakaznik)	Russia	52°18′N 106°25′E	43	43	Wetlands, pastures
Syktyvkar	Russia	61°48′N 51°55′E	35	35	Floodplain meadows
Nizhny Novgorod (Vetluga Village)	Russia	57°46′N 45°27′E	8	8	Abandoned fields

Note

Sample sizes in brackets were not used in the analysis.

To analyze habitat use, we additionally recorded all habitat parameters at pseudo‐absence points, which were placed 100 m to the east of song posts (“absence plots”). We expected these absence plots to be outside of the territory, as distances between nests of 40–100 m were found (Rejmers 1966 in Glutz von Blotzheim & Bauer, 1993). This allowed us to establish preferences for certain habitat features, as opposed to the descriptive notion of habitat use employed when only observational data at presence points are included. Additional pseudo‐absence points were randomly selected in the Amur region, to get a representative sample (*n* > 30) for both burned and unburned wetlands.

### 2.2 Data analysis

All analyses were carried out using R version 3.6.2 (R Development Core Team, 2019). We ran a principal component analysis (PCA) to analyze habitat use including all presence plots via the *prcomp* function. Continuous variables were scaled using the *scale* argument. Only continuous variables that were measured in all study regions were included (Table 2).

**TABLE 2:** Factor loadings of the six principal components based on Yellow‐breasted bunting presence plots and six habitat parameters

Parameter	PC1	PC2	PC3	PC4	PC5	PC6
Vegetation cover	−0.5302	0.1739	−0.0857	0.0689	0.8071	0.1588
Tree cover	−0.5572	−0.0123	0.2736	0.0825	−0.1928	−0.7554
Tree height	−0.5498	0.0372	0.2212	0.1559	−0.4820	0.6251
Shrub cover	0.1923	0.6632	−0.0608	0.7131	−0.0683	−0.0794
Shrub height	0.1029	0.6323	0.5179	−0.5653	0.0283	0.0324
Bare soil cover	0.2420	−0.3586	0.7726	0.3688	0.2714	0.0782

To analyze habitat preferences, generalized linear mixed‐effects models (GLMMs) with a binomial error structure and a logit link were fitted. All study regions with a sufficient sample size of *n* > 10 for both presence and absence plots mapped in the same year were considered for modeling. The resulting dataset contained 240 presence and 328 absence records from three study regions (Amur, Syktyvkar, and Baikal; Table 1). We considered 13 continuous and three categorical variables and first examined them separately in univariate logistic regressions. For continuous variables, we tested whether quadratic relationships improved model fit based on the Akaike information criterion (AIC). Study region was fitted as a random effect in all models. We then built a global model that contained 18 variables that had a significant relationship with presence/absence of the species (at *p* < 0.001) in the univariate models (Table 3).

**TABLE 3:** Effects of environmental parameters on Yellow‐breasted Bunting presence in univariate models

Parameter	*p*‐value	AICc	AUC	*R* ^2^
Vegetation cover	***	693.86	0.69	0.14
Vegetation cover^2^	***	655.58	0.71	0.20
Tree cover	n.s.	778.72	0.54	<0.01
Tree cover^2^	**	770.35	0.54	0.02
Tree height	n.s.	777.81	0.54	<0.01
Tree height^2^	**	773.14	0.53	0.01
Shrub cover	***	634.11	0.85	0.23
Shrub cover^2^	***	521.40	0.87	0.37
Shrub height	***	653.79	0.80	0.20
Shrub height^2^	***	591.34	0.80	0.28
Dwarf shrub cover	***	740.93	0.70	0.06
Dwarf shrub cover^2^	***	714.47	0.71	0.11
Dwarf shrub height	***	728.22	0.70	0.09
Dwarf shrub height^2^	***	697.51	0.73	0.14
Grass cover	**	769.39	0.56	0.02
Grass cover^2^	***	755.36	0.61	0.04
Grass height	***	764.38	0.54	0.03
Grass height^2^	***	727.68	0.64	0.09
Herb cover	***	748.26	0.68	0.05
Herb cover^2^	***	719.60	0.68	0.10
Herb height	n.s.	779.06	0.58	<0.01
Herb height^2^	***	752.92	0.62	0.05
Litter cover	***	760.29	0.60	0.03
Litter cover^2^	n.s.	760.11	0.62	0.04
Litter height	n.s.	778.99	0.54	<0.01
Litter height^2^	.	777.16	0.55	0.01
Bare soil cover	**	768.99	0.46	0.02
Bare soil cover^2^	n.s.	770.99	0.48	0.02
Moisture	***	706.63	0.69	0.13
Fire	n.s.	779.01	0.55	<0.01
Grazing	.	775.85	0.53	0.01
Mowing	n.s.	778.27	0.54	<0.01

Note

Given are respective *p*‐values (***p < 0.001, **p < 0.01, n.s. = not significant, *p* > 0.05), AICc, AUC, and Nagelkerke's pseudo‐*R* ^2^. A superscript 2 indicates quadratic variables (linear terms always included).

We simplified the global model by fitting all possible variable combinations, with a maximum of ten variables in the same model, using the *dredge* function in package MuMIn (Barton, 2015). Only variables that were not strongly correlated were allowed in the same model (Spearman's rho < 0.7). We averaged the selected models using the *model.avg* function based on the AIC for small sample size (AIC_C_). Models with ΔAIC_C_ < 2 were considered to perform equally well (Burnham & Anderson, 2002). The discriminatory ability of the logistic regressions was assessed by the area under the curve (AUC) in ROC plots with function *auc* in package pROC for all models. Nagelkerke's pseudo‐*R* ^2^ was calculated using the *r.squaredLR* function in package MuMIn (Barton, 2015).

For the range‐wide analysis of changes in Yellow‐breasted Bunting habitat parameters, we used data from presence plots of all study sites and fitted zero‐inflated negative binomial GLMMs using the R package glmmTMB (Magnusson et al., 2017). Models were built for each of the numerical habitat parameters (which were available from all study regions) as dependent variable. We fitted latitude and longitude as covariates and study region as random effect. Fixed effects were tested for significance using the *ANOVA* function, and marginal and conditional *R* ^2^ values were calculated using the *r2* function in package performance (Lüdecke et al., 2020).

## 3 RESULTS

**FIGURE 1:**
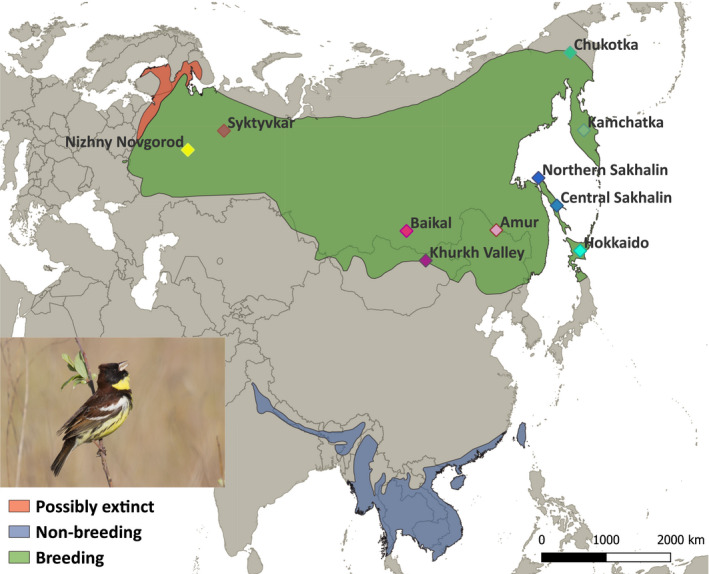
Global breeding and nonbreeding distribution of the Yellow‐breasted Bunting (BirdLife International, 2020) and the location of the study regions. Background map downloaded from https://www.naturalearthdata.com/

We collected information on habitat use at 486 Yellow‐breasted Bunting territories in ten study regions across the breeding range, spanning 20 degrees of latitude and 120 degrees of longitude (Figure 1, Table 1, Table S1). The populations in Nizhny Novgorod and Syktyvkar belong to nominate *E. a. aureola*, whereas all other populations belong to the subspecies *ornata*.

### 3.1 Habitat use

**FIGURE 2:**
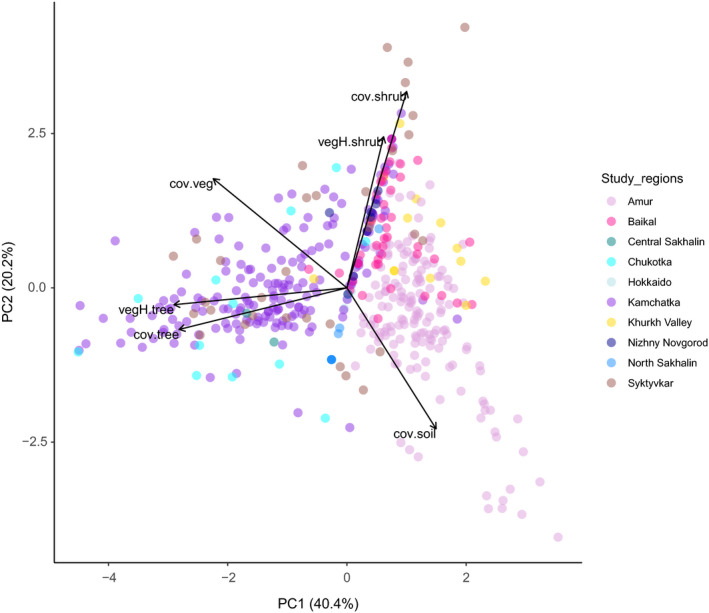
Principal component analysis (PCA) of 486 plots from ten study regions where Yellow‐breasted Buntings were present during the breeding season and correlated habitat parameters (cov.veg = total vegetation cover, cov.tree = tree cover, vegH.tree = mean height of trees, cov.shrub = shrub cover, vegH.shrub = mean height of shrubs, cov.soil = soil cover). Only the first two principal components are shown. Plots that are closer to each other are more similar

The PCA revealed large overlap in habitat parameters between the study regions, and a huge variation within study regions (Figure 2). The first principal component (PC1) was negatively associated with tree cover, tree height, and total vegetation cover and explained 40% of the variance (Table 2). The second principal component was positively associated with shrub cover and shrub height and negatively with bare soil cover, explaining 20% of the variance (Table 2).

### 3.2 Habitat preferences

Our univariate models revealed that all habitat parameters significantly affected the probability of presence of Yellow‐breasted Buntings, with the exception of fire, mowing, and grazing (Table 3). Signs of fire were recorded at 27% of the presences in the Amur region, horse grazing at 74% of the presence plots at Lake Baikal, and mowing at 77% of the presence plots in Syktyvkar (for photos see Figure 4).

We found three multivariate models, which predicted the presence of the Yellow‐breasted Bunting equally well (ΔAIC_C_ < 2) (Table 4). Shrub cover, grass cover, and herb cover were the most important parameters influencing the presence of the Yellow‐breasted Bunting. Those three variables were included in each of the three multivariate models (Table 4).

**TABLE 4:** Summary of the selected multivariate models predicting the presence of the Yellow‐breasted Bunting

	Parameter	AICc	ΔAICc	AUC	*R* ^2^
Model 1	Shrub cover + shrub cover^2^ + dwarf shrub cover + dwarf shrub cover^2^ + grass cover + grass cover^2^ + herb cover + herb cover^2^ + herb height + herb height^2^	478.47	0.00	0.893	0.430
Model 2	Shrub cover + shrub cover^2^ + dwarf shrub cover + dwarf shrub cover^2^ + grass cover + grass cover^2^ + herb cover + herb cover^2^ + grass height + grass height^2^	479.14	0.67	0.892	0.429
Model 3	Shrub cover + shrub cover^2^ + grass cover + grass cover^2^ + grass height + grass height^2^ + herb cover + herb cover^2^ + herb height + herb height^2^	480.42	1.59	0.894	0.428
Null model		777.10	298.63	0.544	

Note

The Akaike information criterion for small size (AIC_C_), ΔAICc, area under curve (AUC), and Nagelkerke's pseudo‐*R* ^2^ are given. A superscript 2 indicates squared terms.

**FIGURE 3:**
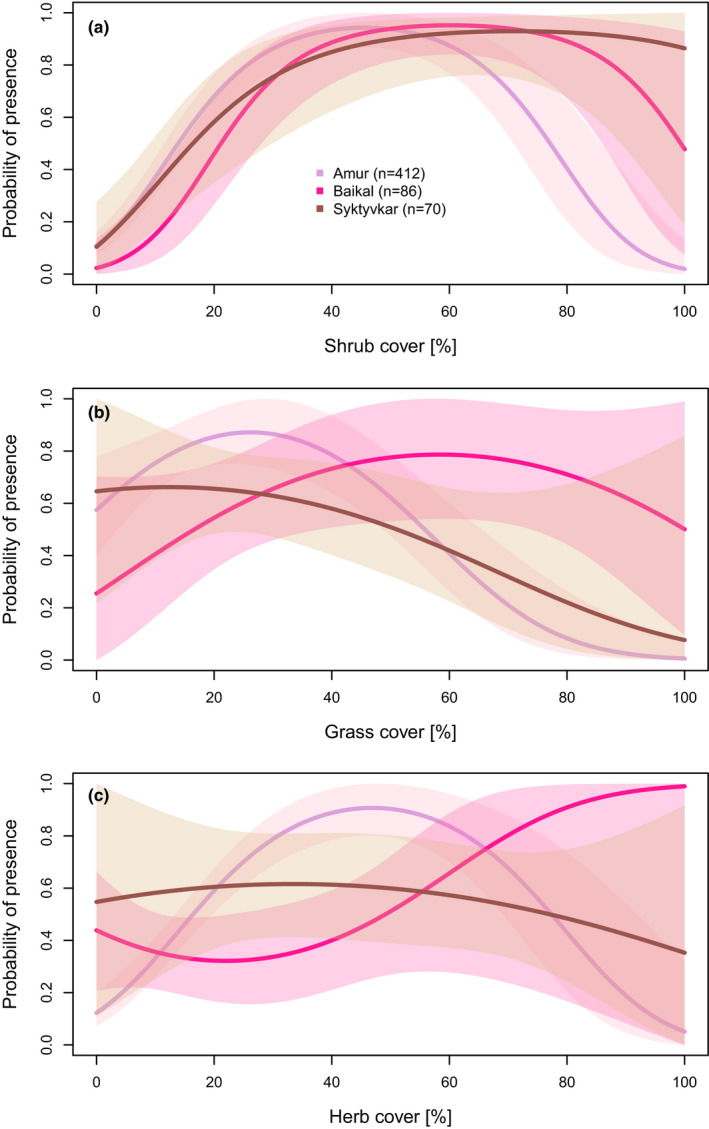
Probabilities of Yellow‐breasted Bunting presence for the most significant habitat parameters (a—shrub cover, b—grass cover, c—herb cover) modeled with data from each of the three study regions separately. The confidence interval (95%) is given in transparent shades

Yellow‐breasted Buntings were recorded at sites with varying shrub cover values (0%–95%), but highest probabilities of presence were predicted at shrub cover values of 40%–55% (Amur), 50%–70% (Lake Baikal), or 60%–90% (Syktyvkar) (Figure 3). Mean shrub cover ranged between 29.9% (±3.6%) in Syktyvkar and 43.4% (±2.8%) at Lake Baikal (Table S1).

Mean grass cover in Yellow‐breasted Bunting territories ranged from 20.8% (±1.2%) at the Amur to 36.1% (±3.2%) at Lake Baikal (Table S1). Mean herb cover in Yellow‐breasted Bunting territories ranged from 20.4% (±2.9%) at Lake Baikal to 39.9% (±3.5%) in Syktyvkar (Table S1).

Three parameters were only included in two of the three multivariate models. These were dwarf shrub cover, grass height, and herb height (Table 4, Table S1).

### 3.3 Range‐wide differences in habitat parameters

We found weak latitudinal and longitudinal effects on the habitat parameters in Yellow‐breasted Bunting presence plots (Table 5). Tree height, shrub cover, grass height, herb cover, and herb height increased with latitude, while soil cover and dwarf shrub height decreased with latitude. Herb cover increased with longitude, while shrub height and dwarf shrub height decreased with longitude.

**TABLE 5:** Effects of latitude and longitude on habitat parameters collected in Yellow‐breasted Bunting territories

Parameter	Latitude	Longitude	$R_{\text{marg}}^{2}$	$R_{\text{cond}}^{2}$
Vegetation cover	*Χ* ^2^ = 0.033, *p* = 0.856, *β* = −0.001	*Χ* ^2^ = 0.007, *p* = 0.931, *β* = 0.000	0.001	0.387
Tree cover	*Χ* ^2^ = 2.635, *p* = 0.105, *β* = 0.093	*Χ* ^2^ = 0.020, *p* = 0.887, *β* = 0.005	0.021	0.541
Tree height	*Χ* ^2^ = 35.947, *p* < 0.001, *β* = 0.134	*Χ* ^2^ = 0.258, *p* = 0.612, *β* = 0.002	0.319	0.589
Shrub cover	*Χ ^2^ = 11.087, p < 0.001, β = 0.099*	*Χ* ^2^ = 0.883, *p* = 0.347, *β* = −0.003	0.157	0.556
Shrub height	*Χ* ^2^ = 1.693, *p* = 0.193, *β* = −0.006	*Χ ^2^ = 7.608, p = 0.006 β = 0.018*	0.182	0.323
Dwarf shrub cover	*Χ* ^2^ = 0.159, *p* = 0.690, *β* = −0.023	*Χ* ^2^ = 1.067, *p* = 0.302, *β* = 0.011	0.043	0.395
Dwarf shrub height	*Χ ^2^ = 24.450, p < 0.001, β = −0.153*	*Χ ^2^ = 22.087, p < 0.001, β = −0.009*	0.648	0.651
Grass cover	*Χ* ^2^ = 3.837, *p* = 0.050, *β* = 0.042	*Χ* ^2^ = 0.406, *p* = 0.524, *β* = 0.003	0.057	0.515
Grass height	*Χ ^2^ = 75.239, p < 0.001, β = 0.163*	*Χ* ^2^ = 3.113, *p* = 0.078, *β* = 0.091	0.305	0.858
Herb cover	*Χ ^2^ = 15.590, p < 0.001, β = −0.146*	*Χ ^2^ = 4.213, p = 0.040, β = −0.001*	0.276	0.668
Herb height	*Χ ^2^ = 11.401, p < 0.001, β = 0.065*	*Χ* ^2^ = 2.184, *p* = 0.140, *β* = 0.003	0.143	0.490
Litter cover	*Χ* ^2^ = 1.394, *p* = 0.238, *β* = −0.080	*Χ* ^2^ = 0.722, *p* = 0.396, *β* = −0.013	0.054	0.795
Bare soil cover	*Χ ^2^ = 5.911, p = 0.015, β = 0.316*	*Χ* ^2^ = 1.477, *p* = 0.224, *β* = 0.002	0.133	0.671

Note

Study region was fitted as random factor. Shown are *Χ* ^2^, *p*‐value (highlighted with gray background if *p* < 0.05), coefficient estimates (*β*), and marginal and conditional *R* ^2^ of negative binomial GLMMs.

## 4 DISCUSSION

We present data on habitat use from a number of breeding populations of the critically endangered Yellow‐breasted Bunting, including two sites in the west of the range, where the species was believed to be extinct (Kamp et al., 2015), and which might have been recolonized recently (Mischenko, 2019) (Figure 1). We found that a wide range of habitats is used (Table 1). The cover of shrubs, herbs, and grasses were the most important of the measured factors to predict the occurrence of the species. We found no evidence for marked differences in habitat use between western and eastern populations, but a strong latitudinal gradient.

**FIGURE 4:**
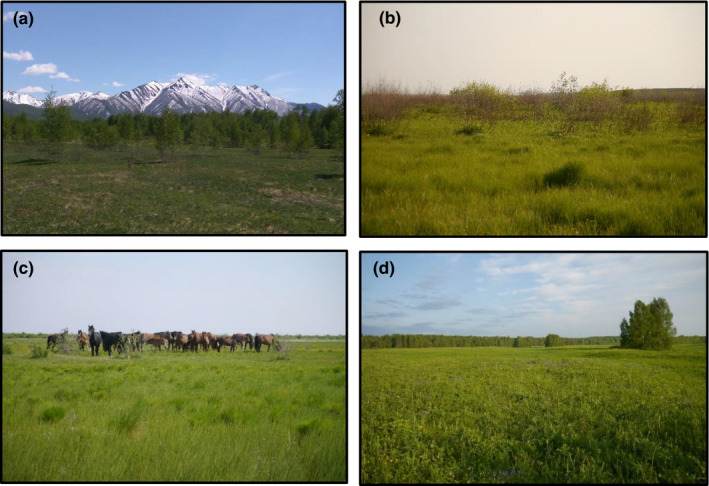
Breeding habitats of Yellow‐breasted Buntings in (a) Kamchatka with loose stands of trees and surrounded by mountains (photo by Y. Gerasimov), (b) burned wetlands in the Amur region with dead willow branches used as perches, (c) wet meadows with few willow shrubs with grazing horses at Lake Baikal, and (d) mowed floodplain meadows in Syktyvkar with small islands of birch trees, which were used as perches (all photos by I. Beermann)

Even within study regions, very different habitats were occupied by Yellow‐breasted Buntings (Figure 4). Much of the variation is explained by differences in the cover and height of trees (Figure 2). Furthermore, we found pronounced differences in the cover and height of shrubs (Figure 2). A wide range of different habitats from treeless wetlands to open forests has also been occupied before the decline (Glutz von Blotzheim & Bauer, 1993).

The mean values of the investigated habitat parameters correspond well to previous studies. For example, a dense grass and herb layer with a height of 30–50 cm was described as a prerequisite for nesting locations (Glutz von Blotzheim & Bauer, 1993), which is similar to the observed values around song posts in our study (mean grass height 50 cm, mean herb height 43 cm). The mean total vegetation cover of 89% in our study is also within the range of previous descriptions (70%–100%) (Glutz von Blotzheim & Bauer, 1993) (Table S1).

A common pattern seems to be the occupation of habitat in early successional stages. Such habitats either result from natural dynamics, for example, forest edges or regularly flooded wetlands, or result from anthropogenic causes, such as man‐made fires in the Amur region, regular mowing near Syktyvkar, horse grazing in the Selenga River delta, or the abandonment of agricultural land in the Nizhny Novgorod region (Table 1). Large‐scale farmland abandonment during and shortly after the breakup of the Soviet Union has likely increased the habitat availability between the 1980s and 2000s, but with ongoing succession and recultivation of abandoned land, the availability of such habitats might decrease (Kamp et al., 2011, 2018).

We found no significant effects of mowing, grazing, or fire on the presence of the Yellow‐breasted Bunting in our models, but this might be explained by low sample size, as those factors were only present in few of the study regions. Nevertheless, changes in traditional mowing regimes could locally lead to habitat loss for the Yellow‐breasted Bunting, for example, in the Syktyvkar region. Earlier, more frequent or more mechanized mowing has been linked to declines in grassland birds in Europe (Green, 1995). However, the area of hay meadows along the Vychegda River in the Syktyvkar region has increased between 2000 and 2019 (G. Nakul, pers. comm.), providing plenty of suitable habitat for the Yellow‐breasted Bunting. In Mongolia, the large increase in livestock numbers may have contributed to widespread habitat loss by promoting short‐grass meadows unsuitable for Yellow‐breasted Buntings, which needs further study in the future (National Statistical Office, 2020). Fire‐mediated habitats, on the contrary, might become increasingly available in the future, given the current increase in fire frequency and extent (Flannigan et al., 2009). More frequent fires have been observed in wetlands of the Amur region, where one of the largest known breeding populations of the Yellow‐breasted Bunting is thriving (Heim et al., 2019; Richter et al., 2020). But since our study regions were not selected randomly, accessible sites near human habitation shaped by anthropogenic drivers of succession might be overrepresented in our survey.

Our data were only collected at song posts, and the species' preferences for foraging or nesting habitats might be narrower. But given that song posts are often very close to nesting territories (Glutz von Blotzheim & Bauer, 1993), we feel confident that we sampled representative habitats.

Nevertheless, the large variation in habitat use, even within study regions, possibly indicates that the niches of the survived Yellow‐breasted Bunting populations might still be relatively wide. Our results demonstrate that this species is able to use grass‐ and shrub‐dominated habitats with a wide range of vegetation structures.

Using presence–absence data from three study regions, we found that the cover of shrubs, herbs, and grasses were the best of our measured variables to predict the occurrence of the Yellow‐breasted Bunting. Highest probabilities of occurrence were found at sites with an intermediate shrub cover of 40%–70% (Figure 3). The cover of willow shrubs was also found to best predict the presence of the species at a breeding site in the Amur region, using remote sensing data (Richter et al., 2020). The very high probabilities of presence with increasing shrub cover predicted for Syktyvkar might stem from the rather low sample size from this study region, including very few plots with high shrub cover values, and might be unrealistic.

The optima of the other two main habitat parameters differed significantly between the study regions (Figure 3). These regional differences must be considered when modeling the distribution of the Yellow‐breasted Bunting on a range‐wide scale.

The observed longitudinal differences in breeding habitat parameters are marginal, but more obvious are latitudinal changes (Table 5). In the south of the range, more open habitats with low shrubs are occupied, while in the north, habitats comprise higher trees and denser shrub cover. This might be explained by different habitat preferences of northern and southern populations, or by differences in habitat availability (e.g., different levels of grass and shrub heights in different latitudes). We found no evidence for possible differences in habitat use between the two subspecies (Glutz von Blotzheim & Bauer, 1993). Our results must be interpreted with caution, since our dataset is strongly biased toward the more numerous eastern populations, and since we collected data only after the decline happened.

## 5 CONCLUSIONS

By compiling a large dataset on habitat use on a very wide spatial scale, we found huge variation in habitat use among populations of the critically endangered Yellow‐breasted Bunting after its decline. This flexibility is a feature, which has probably allowed the species to spread to the west quickly in the 19th century (Mischenko, 2019), and which might allow the species to recover fast if the current limiting factors can be eliminated. Our results provide important information for future studies to estimate suitable habitat cover at larger spatial scales (from regional to landscape scales), and to model the distribution of the Yellow‐breasted Bunting on a range‐wide scale. We argue that further studies should also investigate habitat use during the nonbreeding season, given that Yellow‐breasted Buntings are suspected to face more threats at stopovers and wintering grounds (Heim et al., 2021).

## CONFLICT OF INTEREST

None declared.

## AUTHOR CONTRIBUTION

**Ilka Beermann:** Conceptualization (supporting); Data curation (lead); Formal analysis (lead); Investigation (equal); Visualization (lead); Writing‐original draft (equal). **Alexander Thomas:** Conceptualization (supporting); Data curation (supporting); Formal analysis (supporting); Investigation (equal); Writing‐review & editing (supporting). **Yury Anisimov:** Investigation (equal). **Marc Bastardot:** Investigation (equal). **Nyambayar Batbayar:** Investigation (equal); Writing‐review & editing (supporting). **Davaasuren Batmunkh:** Investigation (equal); Writing‐review & editing (supporting). **Yury Gerasimov:** Investigation (equal). **Makoto Hasebe:** Investigation (equal); Writing‐review & editing (supporting). **Gleb Nakul:** Investigation (equal). **Jugdernamjil Nergui:** Investigation (equal). **Pavel Ktitorov:** Investigation (equal); Writing‐review & editing (supporting). **Olga Kulikova:** Investigation (equal); Visualization (supporting); Writing‐review & editing (supporting). **Wieland Heim:** Conceptualization (lead); Data curation (equal); Formal analysis (supporting); Investigation (equal); Methodology (equal); Visualization (supporting); Writing‐original draft (equal); Writing‐review & editing (lead).

## Supporting information

Table S1

## Data Availability

Habitat parameter data are available on Dryad (https://doi.org/10.5061/dryad.0rxwdbs0g).
