# Supplementary material for: Range‐wide breeding habitat use of the critically endangered Yellow‐breasted Bunting Emberiza aureola after population collapse
Source: Ecol Evol. 2021 May 18;11(13):8410–9. doi: 10.1002/ece3.7668 (PMC8258230; doi:10.1002/ece3.7668)
Supplement: Supplementary file 1 — Table S1 [file ECE3-11-8410-s001.docx]

|  | **Total** | | **Chukotka** | **Kamchatka** | | **Sakhalin** | | **Hokkaido** | **Mongolia** | | **Amur** | | **Baikal** | | **Syktyvkar** | | **Nizhny Novgorod** | |
| --- | --- | --- | --- | --- | --- | --- | --- | --- | --- | --- | --- | --- | --- | --- | --- | --- | --- | --- |
|  |  |  |  |  |  | **north** | **south** |  |  |  |  |  |  |  |  |  |  |  |
| **variable** | **(**N_P_ **=348)** | **(**N_A_ **=459)** | **(**N_P_ **=12)** | **(**N_P_ **=190)** | **(**N_A_ **=31)^C^** | **(**N_P_ **=12)** | **(**N_P_ **=2)** | **(**N_P_ **=11)** | **(**N_P_ **=14)** | **(**N_A_ **=10)** | **(**N_P_ **=162)** | **(**N_A_ **=250)** | **(**N_P_ **=43)** | **(**N_A_ **=43)** | **(**N_P_ **=35)** | **(**N_A_ **=35)** | **(**N_P_ **=8)** | **(**N_A_ **=8)** |
| cov.veg [%] | 88.61 (± 0.69) | 62.94 (± 1.52) | 96.25 (± 2.55) | 99.4 (± 0.27) | 91.94 (± 3.63) | 100.0 (± 0.00) | 100.0 (± 0.00) | 100.0 (± 0.00) | 83.07 (± 3.39) | 71.50  (± 8.73) | 72.44  (± 1.04) | 54.14  (± 1.66) | 91.05  (± 1.99) | 68.56  (± 4.92) | 90.69  (± 1.99) | 83.69  (± 4.23) | 100.0 (± 0.00) | 93.75  (± 6.25) |
| cov.tree [%] | 8.76  (± 0.74) | 3.18  (± 0.64) | 33.75 (± 7.49) | 16.87  (± 1.40) | 19.03 (± 4.69) | 0.00 (± 0.00) | 25.0 (± 5.00) | 0.0  (± 0.00) | 4.29  (± 4.29) | 4.00  (± 3.48) | 0.16  (± 0.11) | 1.44  (± 0.58) | 0.53  (± 0.47) | 1.05  (± 0.66) | 14.51  (± 3.00) | 4.57  (± 2.37) | 0.62 (± 0.62) | 0.62 (± 0.62) |
| vegH.tree [cm] | 335.52  (± 22.55) | 86.73  (± 15.69) | 691.67 (± 111.78) | 763.07  (± 33.16) | 412.90 (± 71.65) | 0.0 (± 0.00) | 400.0 (± 100.0) | 0.0  (± 0.00) | 21.43  (± 21.43) | 82.00  (± 54.99) | 3.39  (± 2.36) | 34.34  (± 13.80) | 42.00  (± 29.11) | 74.00  (± 41.34) | 665.83  (± 88.54) | 255.56  (±128.14) | 400.00 NA | 400.00 NA |
| cov.shrub [%] | 30.09  (± 0.99) | 11.52  (± 1.05) | 25.92 (± 8.34) | 27.79  (± 1.58) | 23.39 (± 4.01) | 6.67 (± 5.78) | 3.00 (± 2.00) | 6.36  (± 2.62) | 55.93  (± 4.72) | 9.50  (± 3.02) | 30.85  (± 1.52) | 11.18  (± 1.35) | 43.37  (± 2.76) | 9.63  (± 2.92) | 29.91  (± 3.60) | 4.66  (± 2.08) | 34.38 (± 6.30) | 18.75 (± 5.96) |
| vegH.shrub [cm] | 183.26  (± 4.80) | 95.19  (± 6.78) | 129.17 (± 24.01) | 175.90  (± 5.03) | 121.94 (± 11.39) | 15.83 (± 8.48) | 150.0 (± 50.0) | 115.00  (± 28.92) | 257.86 (± 20.56) | 162.50  (± 46.89) | 162.93  (± 3.11) | 81.06  (± 7.68) | 238.95  (± 13.01) | 112.97  (± 22.88) | 313.28  (± 49.06) | 97.00  (± 41.23) | 243.75 (± 21.95) | 298.57 (± 30.97) |
| cov.dshrub [%] | 6.80  (± 0.57) | 3.21  (± 0.41) | *NA* | 5.33  (± 1.00) | 0.81 (± 0.57) | *NA* | *NA* | 16.88  (± 5.50) | 8.00  (± 5.29) | 1.22  (± 1.10) | 10.71  (± 0.85) | 4.26  (± 0.57) | 0.30  (± 0.12) | 0.19  (± 0.13) | 1.34  (± 0.86) | 0.40  (± 0.20) | 9.38 (± 3.33) | 10.62 (± 4.77) |

**Table S1**: Mean and standard error (se) of presence data (N_P_) for all ten study regions. For Mongolia, Amur, Baikal and Syktyvkar mean and se are given for absence data (N_A_), too. Factorial parameters are segmented as follows: * moisture in four classes [0 = completely dry, 1 = moist or wet, 2 = waterlogged, 3 = standing open water or flooded soil], ** fire, grazing and mowing in two classes [0 = no event happened at the plot, 1 = an event happened at the plot]. The superscript C indicates that absence data in Kamchatka were collected one year after collection of presence data.

|  | **Total** | | **Chukotka** | **Kamchatka** | | **Sakhalin** | | **Hokkaido** | **Mongolia** | | **Amur** | | **Baikal** | | **Syktyvkar** | | **Nizhny Novgorod** | |
| --- | --- | --- | --- | --- | --- | --- | --- | --- | --- | --- | --- | --- | --- | --- | --- | --- | --- | --- |
|  |  |  |  |  |  | **north** | **south** |  |  |  |  |  |  |  |  |  |  |  |
| **variable** | **(**N_P_ **=348)** | **(**N_A_ **=459)** | **(**N_P_ **=12)** | **(**N_P_ **=190)** | **(**N_A_ **=31)^C^** | **(**N_P_ **=12)** | **(**N_P_ **=2)** | **(**N_P_ **=11)** | **(**N_P_ **=14)** | **(**N_A_ **=10)** | **(**N_P_ **=162)** | **(**N_A_ **=250)** | **(**N_P_ **=43)** | **(**N_A_ **=43)** | **(**N_P_ **=35)** | **(**N_A_ **=35)** | **(**N_P_ **=8)** | **(**N_A_ **=8)** |
| vegH. dshrub [cm] | 37.99 (± 1.66) | 19.49  (± 1.67) | *NA* | 27.45  (± 1.93) | 2.58 (± 1.79) | *NA* | *NA* | 45.00  (± 3.27) | 71.43 (± 13.88) | 16.67  (± 11.06) | 42.99  (± 1.94) | 21.38  (± 1.90) | 15.97  (± 5.49) | 6.85  (± 3.84) | 28.46  (± 6.80) | 18.33  (± 6.56) | 84.29 (± 5.28) | 93.33 (± 3.33) |
| cov.grass [%] | 32.60  (± 1.16) | 31.22  (± 1.30) | 57.08 (± 8.76) | 32.52  (± 1.82) | 31.29 (± 5.12) | 90.17 (± 8.19) | 100.0 (± 0.0) | 35.62  (± 7.22) | 71.64  (± 4.91) | 68.30  (± 8.03) | 20.77  (± 1.17) | 28.61  (± 1.55) | 36.05 (± 3.24) | 31.40  (± 3.57) | 27.20  (± 2.33) | 34.51  (± 3.25) | 68.12 (± 6.88) | 50.62 (± 10.96) |
| vegH.grass [cm] | 50.48  (± 1.95) | 45.93  (± 1.94) | 57.92 (± 7.57) | 67.87  (± 4.31) | 61.94 (± 4.85) | 120.83 (± 11.31) | 90.0 (± 10.0) | 50.00  (± 0.00) | 9.07  (± 1.91) | 8.00 (± 1.54) | 34.66  (± 0.98) | 49.11  (± 2.65) | 38.37  (± 3.48) | 31.43  (± 4.08) | 37.57  (± 1.57) | 38.43  (± 1.67) | 51.88 (± 4.99) | 40.00 (± 4.63) |
| cov.herb [%] | 29.66  (± 1.01) | 20.14  (± 1.09) | *NA* | 31.30  (± 1.74) | 28.61 (± 4.47) | *NA* | *NA* | 55.45  (± 7.79) | 5.36  (± 3.57) | 4.50  (± 2.31) | 28.61  (± 1.34) | 16.52  (± 1.17) | 20.37  (± 2.88) | 16.26 (± 2.06) | 39.86  (± 3.49) | 40.74  (± 3.73) | 24.38 (± 6.58) | 41.88 (± 12.10) |
| vegH.herb [cm] | 42.46  (± 1.28) | 31.31  (± 1.36) | *NA* | 59.83  (± 2.26) | 44.35 (± 6.09) | *NA* | *NA* | 35.45  (± 2.82) | 24.88 (± 14.42) | 3.00  (± 1.66) | 31.58  (± 1.15) | 31.53  (± 1.69) | 18.12  (± 1.41) | 19.02  (± 2.10) | 37.14  (± 2.49) | 33.86  (± 2.72) | 61.25  (± 4.70) | 59.38 (± 9.08) |
| cov.litt [%] | 71.97  (± 1.98) | 60.07  (± 2.08) | 82.92 (± 8.45) | *NA* | 0.00 (± 0.00) | 23.00 (± 7.58) | 100.0 (± 0.0) | 16.67  (± 5.27) | 23.21  (± 7.36) | 13.00  (± 5.32) | 83.83  (± 1.97) | 72.47  (± 2.23) | 72.28  (± 5.20) | 54.86  (± 5.87) | 61.17  (± 6.33) | 44.69  (± 6.61) | 75.00 (± 3.27) | 59.38 (± 10.20) |
| h.litt [cm] | 8.06  (± 0.89) | 5.13  (± 0.71) | 1.08 (± 0.08) | 66.67 (± 11.74) | 0.00 (± 0.00) | 48.33 (± 9.44) | 1.00 (± 0.00) | 3.18  (± 0.76) | 3.14  (± 0.71) | 2.30  (± 0.58) | 3.69  (± 0.30) | 4.55  (± 0.34) | 12.60  (± 1.87) | 16.62  (± 5.74) | 6.77  (± 1.66) | 1.63  (± 0.15) | 1.00 (± 0.00) | 1.14 (± 0.14) |
| cov.soil [%] | 5.86  (± 0.70) | 16.07  (± 1.44) | 3.75 (± 2.55) | 1.54  (± 0.41) | 14.71 (± 5.89) | 0.42 (± 0.42) | 0.00 (± 0.00) | 0.00  (± 0.00) | 14.79  (± 3.26) | 19.00  (± 7.01) | 12.70  (± 1.87) | 19.43  (± 1.92) | 4.60  (± 1.03) | 7.93  (± 1.88) | 1.57  (± 0.56) | 5.60  (± 2.95) | 0.00 (± 0.00) | 0.00 (± 0.00) |

|  | **Total** | | **Chukotka** | **Kamchatka** | | **Sakhalin** | | **Hokkaido** | **Mongolia** | | **Amur** | | **Baikal** | | **Syktyvkar** | | **Nizhny Novgorod** | |
| --- | --- | --- | --- | --- | --- | --- | --- | --- | --- | --- | --- | --- | --- | --- | --- | --- | --- | --- |
|  |  |  |  |  |  | **north** | **south** |  |  |  |  |  |  |  |  |  |  |  |
| **variable** | **(**N_P_ **=348)** | **(**N_A_ **=459)** | **(**N_P_ **=12)** | **(**N_P_ **=190)** | **(**N_A_ **=31)^C^** | **(**N_P_ **=12)** | **(**N_P_ **=2)** | **(**N_P_ **=11)** | **(**N_P_ **=14)** | **(**N_A_ **=10)** | **(**N_P_ **=162)** | **(**N_A_ **=250)** | **(**N_P_ **=43)** | **(**N_A_ **=43)** | **(**N_P_ **=35)** | **(**N_A_ **=35)** | **(**N_P_ **=8)** | **(**N_A_ **=8)** |
| moisture * | 0.97  (± 0.04) | 1.18  (± 0.06) | 1.08 (± 0.15) | 0.86  (± 0.07) | 1.14 (± 0.22) | 1.08 (± 0.08) | 1.00 (± 0.00) | 1.00  (± 0.00) | 1.36  (± 0.27) | 0.70  (± 0.15) | 0.84  (± 0.04) | 1.04  (± 0.07) | 1.51  (± 0.11) | 2.02  (± 0.14) | 1.26  (± 0.11) | 1.20  (± 0.11) | 0.88 (± 0.12) | 1.38 (± 0.37) |
| fire ** | 0.09  (± 0.01) | 0.16  (± 0.02) | 0.00 (± 0.00) | 0.00  (± 0.00) | 0.00 (± 0.00) | 0.00 (± 0.00) | 0.00 (± 0.00) | 0.00  (± 0.00) | 0.00  (± 0.00) | 0.00  (± 0.00) | 0.27  (± 0.03) | 0.24  (± 0.03) | 0.05  (± 0.03) | 0.05  (± 0.03) | 0.00  (± 0.00) | 0.00  (± 0.00) | 0.00 (± 0.00) | 0.00 (± 0.00) |
| grazing ** | 0.11  (± 0.01) | 0.11  (± 0.02) | 0.00 (± 0.00) | 0.04  (± 0.01) | 0.06 (± 0.04) | 0.00 (± 0.00) | 0.00 (± 0.00) | 0.00  (± 0.00) | 0.93  (± 0.07) | 1.00  (± 0.00) | 0.01  (± 0.01) | 0.02  (± 0.01) | 0.74  (± 0.07) | 0.53  (± 0.08) | 0.00  (± 0.00) | 0.00  (± 0.00) | 0.00 (± 0.00) | 0.00 (± 0.00) |
| mowing ** | 0.06  (± 0.01) | 0.08  (± 0.01) | *NA* | 0.00  (± 0.00) | 0.00 (± 0.00) | 0.00 (± 0.00) | 0.00 (± 0.00) | 0.00  (± 0.00) | 0.00  (± 0.00) | 0.00  (± 0.00) | 0.00  (± 0.00) | 0.00  (± 0.00) | 0.00  (± 0.00) | 0.00  (± 0.00) | 0.77  (± 0.07) | 0.77  (± 0.07) | 0.00 (± 0.00) | 0.00 (± 0.00) |
